# Preparation of Alginate Oligosaccharides by Autoclaving Pretreatment Combined with Enzymatic Method

**DOI:** 10.3390/md24040127

**Published:** 2026-03-30

**Authors:** Feiyu Niu, Ziqiang Gu, Zihan Yu, Zhi Bao, Jichao Li, Peng Yang, Dongyu Li, Haijin Mou, Changliang Zhu

**Affiliations:** 1 College of Food Science and Engineering, Ocean University of China, Qingdao 266003, China; 15686290590@163.com (F.N.); yzh963769256@163.com (Z.Y.); 18388177701@163.com (Z.B.); 13969597234@163.com (J.L.); 15171165896@163.com (P.Y.); ldy1516@ouc.edu.cn (D.L.); 2 School of Food Science and Engineering, South China University of Technology, Guangzhou 510641, China; ziqianggu1568@163.com; 3 Dongguan Hsu Chi Food Co., Ltd., Dongguan 523118, China

**Keywords:** high-pressure pretreatment, sodium alginate, alginate oligosaccharide, autoclaving, enzymolysis process

## Abstract

The enzymatic method is the primary focus for alginate oligosaccharide (AOS) production. However, the high viscosity of sodium alginate (SA) substrate often limits enzymatic efficiency. Pretreatment strategies aimed at reducing SA viscosity offer a promising and innovative solution to enhance process efficiency. This study compared the effects of three pretreatment methods—high-pressure vapor (HP-v), high-pressure solution (HP-s), and atmospheric-pressure air (AP-a)—on the physicochemical properties of SA. These pretreatments reduced SA viscosity and induced visible color changes in the order HP-v > HP-s > AP-a. Additionally, the effects of high-pressure treatments on molecular weight, M/G ratio, and chemical structure of SA were analyzed, confirming the feasibility of pretreatment-assisted enzymolysis. Molecular weight distribution and ESI-MS analysis of AOS after enzymolysis demonstrated that brief HP-v treatment maximizes the catalytic potential of alginate lyase, facilitating efficient AOS production without altering its structural characteristics.

## 1. Introduction

Sodium alginate (SA) is a kind of natural acidic polysaccharide found in the cell walls of brown seaweeds, which can also be secreted by microorganisms such as *Azotobacter vinelandii* and *Pseudomonas aeruginosa* [1,2]. This unbranched chain copolymer consists of β-D-mannuronic acid (M) and its C5 epimer α-L-guluronic acid (G) in different proportions, which are connected by β-1,4-glycosidic bonds in a non-repeating block manner [3]. The properties of SA are highly dependent on its sources and the extraction methods employed, which influence the ratio and distribution of M and G units [4,5]. However, the physiological activity of SA has been significantly limited due to its high molar mass, long molecular chains and poor water solubility [6,7]. Alginate oligosaccharide (AOS) and low-molecular-weight sodium alginate (LMWSA), prepared by the SA degradation, overcome the solubility and viscosity issues and show a variety of beneficial biological activities over high-molecular-weight sodium alginate (HMWSA) [8]. From the perspective of molecular chain, the space structure of HMWSA is totally complex, which makes many active sites difficult to be fully exposed and easy to be covered. In contrast, AOS and LMWSA can enter biological tissues more easily because of the small volume, resulting in fast molecular motion [9,10].

Given the increasing significance of AOS and LMWSA in high-value applications, various preparation methods have been used to explore the potential of diverse AOS products [11]. Compared with a variety of physical and chemical methods, the enzymatic method is conducive to expanding industrial and commercial scale. It allows precise control of reaction conditions based on the properties of alginate lyase, enabling the production of AOS and LMWSA with specific monomer sequences and oligomer compositions. The monomer sequence and structural composition of these products are easier to analyze, which determine active biological functions and beneficial health effects [12]. Current studies primarily focus on improving enzymolysis efficiency through three aspects: alginate lyase engineering [13], fermentation processes for alginate lyase [14] and the alginate enzymolysis process [15]. However, limited attention has been given to addressing the practical challenges in production. Typically, high-molecular-weight SA serves as the substrate for AOS enzymatic production. Upon dissolution in water, SA forms a highly viscous colloid that can expand up to ten times its original volume due to water absorption [6]. This high viscosity hampers efficient enzymolysis by causing slow dissolution, uneven substrate distribution, and limited enzyme/substrate contact. Adjusting the pH according to enzyme properties becomes particularly challenging under these conditions. Additionally, the significant shearing force during stirring generates excessive heat [16], and altering the temperature of the enzymolysis substrate adversely affects the activity of alginate lyase, further constraining enzymolysis efficiency in industrial applications.

Therefore, overcoming the challenges brought by high substrate concentration is crucial for enhancing the efficiency of AOS production. Currently, employing appropriate pretreatment methods to reduce substrate viscosity allows higher substrate concentrations, which presents a promising strategy. Compared with chemical treatment, the physical method is a preferable option due to the straightforward operation, low cost, and absence of chemical reagent introduction. High-pressure treatment, particularly when implemented using an autoclave, has emerged as a prominent physical method owing to its practicality and accessibility [17]. This technique can enhance production efficiency by disrupting hydrogen bonds and hydrophobic forces, thereby improving the mass transfer of solvents into raw materials and increasing the soluble constituents [18]. Furthermore, high-pressure treatments are typically conducted in a closed system, preventing solvent evaporation and offering environmental benefits [19]. Several studies have reported the application of high-pressure treatments in the extraction and processing of polysaccharides. Su et al. [20] demonstrated that the water-soluble polysaccharides prepared from *Grifola frondosa* at 121 °C yielded higher quantities and contained significantly higher levels of (1→3, 1→6)-β-D-glucans compared to those prepared at 70 °C and 100 °C. These findings suggest that the autoclave-based extraction is an effective strategy for rapid and high-quality preparation of water-soluble polysaccharides. Similarly, polysaccharides in *Pleurotus eryngii* mushroom were extracted in an autoclave at 121 °C for 1 h, resulting in its fractions comprising at least four distinct α- and β-glucans, which improved the content of linear (1→3)-α-D-glucans [21]. Moreover, it has been reported that the viscosity of fucoidan aqueous solution can be reduced by 91% after hydrolysis using an autoclave reactor at 121 °C for 40 min [22]. These studies underscore the potential of high-pressure treatments in polysaccharide modification and oligosaccharide preparation. However, little research is available about the degradation of SA by high-pressure treatments, and the pretreatment methods and approaches such as auxiliary enzymolysis are rarely reported.

The realization of high-pressure conditions in autoclave is often accompanied by high temperature. In order to explore the influence of high-pressure treatment on SA, the changes of SA after atmospheric hot air treatment, high-pressure solution treatment and high-pressure vapor treatment were compared in this study. It was hypothesized that by controlling the treatment conditions of SA at high temperature in an appropriate medium, a great viscosity reduction effect could be achieved, so as to serve as an auxiliary way for enzymolysis of SA to improve efficiency indirectly. Key questions addressed include whether the specific pretreatments could induce any changes in the chemical structure of SA and whether such changes are acceptable for subsequent applications. To explore these issues, this study focused on evaluating the degradation effects of SA in various treatment media, particularly the impact of high-pressure treatments on SA’s structure and depolymerization. Additionally, the impact of pretreatment on SA enzymolysis was analyzed to assess its potential to integrate physical methods with enzymatic processes, providing a novel perspective for AOS preparation.

## 2. Results and Discussion

### 2.1. Changes in Performance of SA with Different Heating Media

#### 2.1.1. Changes in Viscosity of SA

**Figure 1:**
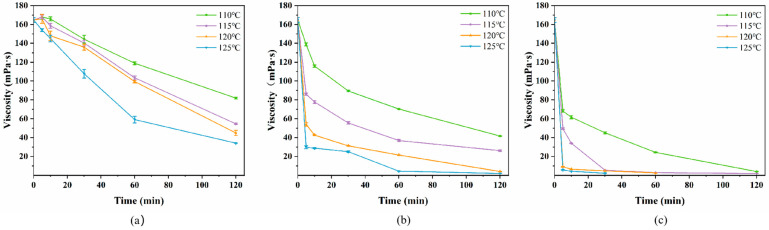
Changes in viscosity of SA under different heating media: (**a**) AP-a treatment; (**b**) HP-s treatment; (**c**) HP-v treatment. Note: The viscosity of sodium alginate solution treated at 120 °C for 120 min and that at 125 °C for 120 min were lower than the range of the viscometer.

The viscosity of SA solution is mainly influenced by the polymerization degree and concentration of polysaccharide, and the decrease in viscosity is indicative of the depolymerization of polysaccharide [23]. As shown in Figure 1a, the viscosity of SA subjected to atmospheric hot air exhibited a decreasing trend with prolonged treatment time, with more pronounced reductions observed at elevated temperatures (*p* < 0.05). Notably, in all groups except for the AP-a treatment at 125 °C, the viscosity displayed a slight increase at 5 min. This anomaly was likely attributed to the limited thermal degradation occurring at the early stages of heating, coupled with the rapid evaporation of water, which temporarily concentrates the solution. The treatment at 125 °C for 120 min demonstrated the most pronounced viscosity reduction, from 164.77 mPa·s to 34.08 mPa·s. Nonetheless, the AP-a treatment in limited experimental conditions did not reach a plateau in viscosity reduction.

After high-pressure treatments, the viscosity changes of SA followed a similar trend to that observed with the AP-a treatment. However, the viscosity reduction effects of both HP-s and HP-v treatments were significantly more effective than that of the AP-a treatment. The AP-a treatment required a longer duration to achieve the desired viscosity reduction, whereas Figure 1b,c show that high-pressure treatments could substantially reduce viscosity more rapidly. Taking the treatment at 120 °C for 5 min as an example, the viscosity of SA in the HP-s treatment decreased from the initial 164.67 mPa·s to 54.00 mPa·s, with a decrease of 67%, and that in the HP-v treatment decreased to 9.83 mPa·s, with a decrease of 94%, both of which were more effective than that in the AP-a treatment at 125 °C for 60 min (59.07 mPa·s). Notably, the HP-v treatment proved particularly effective, reducing the viscosity of SA by approximately 58% following autoclaving at 110 °C for 5 min. The viscosity reduction effect was comparable at 120 °C and 125 °C, but beyond 120 °C, further increases in treatment temperature yielded only marginal improvements in viscosity reduction. The depolymerization of SA under hydrothermal treatment (180–240 °C) was examined, which initially involved the release of mannuronic acid, followed by guluronic acid. Monosaccharides decomposed to form water-soluble acids and other solids and gases as the reaction progressed [24]. Consequently, prolonged high-temperature treatment might result in the formation of substances other than mannuronic acid and guluronic acid, affecting the uniformity and stability of polysaccharide products. Furthermore, taking energy consumption during the treatment process into account, short-duration treatments of SA were more advantageous for enhancing industrial production efficiency.

#### 2.1.2. Color Changes of SA

During treatments of SA with the aforementioned three media, a reduction in viscosity was accompanied by noticeable color changes, consistent with observations in previously reported physical processing methods [23,25]. With increasing treatment intensity, the color of SA gradually turned brown. To further evaluate this phenomenon, the color changes of SA solutions were analyzed under two specific conditions: after 5 min treatment at varying temperatures and after treatments of differing durations at 120 °C.

**Figure 2:**
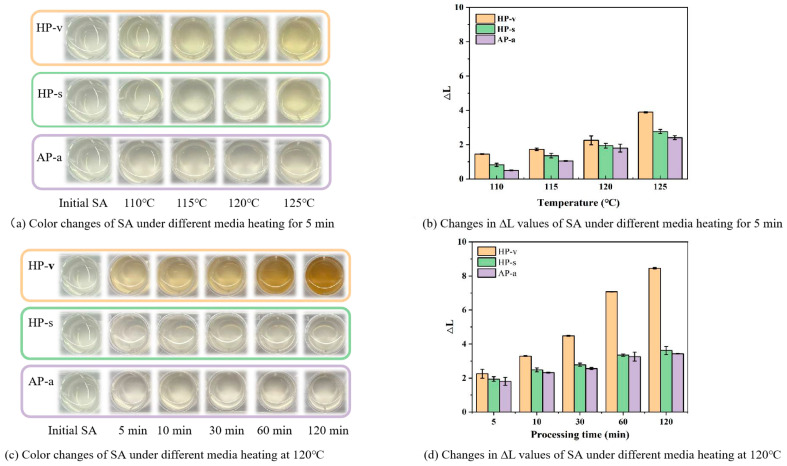
Color changes of SA under different heating media conditions.

As shown in Figure 2a, compared to the original colorless and transparent SA solution, the SA solutions from all treatment groups treated for 5 min gradually turned yellow and brown as the temperature increased from 110 °C to 125 °C. Among the three treatment groups, the HP-v treatment exhibited the most significant color change. Furthermore, the appearance of SA solutions underwent the most noticeable color transformation when treated at 125 °C. Low ∆L values were observed up to 120 °C in Figure 2b, indicating no obvious decrease in the brightness of SA solutions. Figure 2c illustrates the color changes in the SA solutions subjected to different heating media at 120 °C. As the processing time extended, the degree of browning in the SA solutions became more pronounced, which was also reflected in the ∆L values shown in Figure 2d. The HP-v treatment exhibited the most significant color change, with the solution gradually turning brown. The HP-s treatment also exhibited an increase in color intensity to some extent, while the AP-a treatment showed the least color change. Several studies have documented color changes in SA during the degradation process. The SA color also shifted to brown with increasing γ-irradiation doses, and the highest irradiation dose without causing significant browning in the alginate solution was 100 kGy [23]. Additionally, intense browning was observed in a 0.9% alginate solution when the plasma treatment duration exceeded 45 min, with no color change shown at lower concentrations and shorter treatment time [26].

In addition to color changes observed during physical processing, the color also intensifies during the enzymolysis of SA. It is inevitable that undehydrated AOS products reflect the natural hues of yellow, brown, and other algae. Therefore, minor color changes resulting from pretreatment methods aimed at achieving the desired viscosity reduction are deemed acceptable. Based on the above results, the suitable treatment condition to prevent significant browning of the SA solution was heating for 5 min, which would not cause excessive color changes.

**Figure 3:**
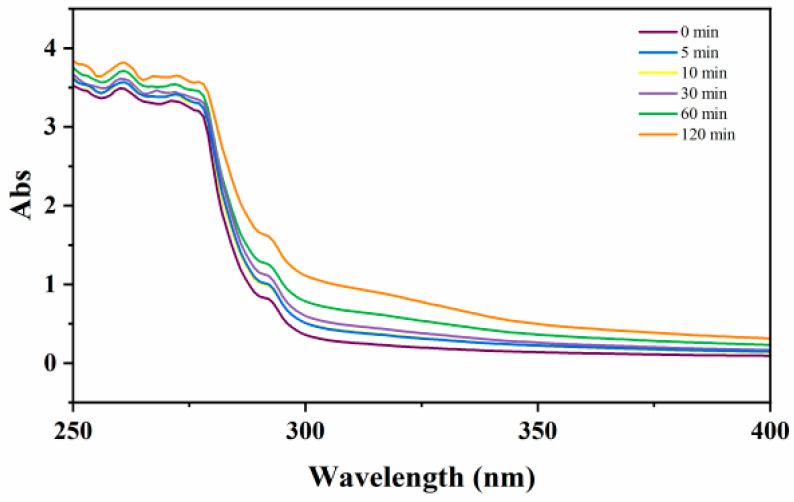
UV-vis adsorption spectra of SA treated at 125 °C in HP-v treatment.

It was reported that a new characteristic absorption band appeared in the UV-vis spectra around 265 nm of SA solutions as the color turned yellow-brown [25,27]. Wasikiewicz et al. [28] indicated the specific absorption band at 265 nm emerged and intensified with exposure time during the ultraviolet degradation of SA. During the degradation of SA by Co^60^ gamma rays, the absorption peak intensity at 265 nm increased with dose, which was attributed to double bonds of alginate formed after main chain scission [27]. Given the similar discoloration phenomenon in this study, the UV-vis absorption spectra of SA solution in HP-v treatment at 125 °C which showed the most obvious color changes were determined. As shown in Figure 3, the waveform moved up as a whole with the increase in processing time, but no new characteristic absorption band at 265 nm appeared. This suggested that the color changes of SA under autoclaving were not linked to the formation of unsaturated double bonds during degradation. Moreover, browning of solutions containing carbohydrates, which are heated at very high temperatures, could be due to complex cascade reactions involving consecutive water elimination, condensation and oligomerization processes, that allow the conversion of monosaccharide residues into aromatic species with extended π-conjugation [29,30]. These moieties could have such high ε values at visible wavelengths to make the solution brown, even if such a dehydration process regards only a very limited amount of the monosaccharides residues along the polysaccharide backbone. The correlation between SA browning and absorption in the visible region of the UV-visible spectrum will be validated in our further studies.

### 2.2. Structural Changes of SA After High-Pressure Treatment

The AP-a treatment conformed to the conventional thermal degradation model of SA, a model whose mechanism and characteristics have been extensively discussed [30,31,32,33]. However, scant information exists regarding the degradation of SA via autoclaving, especially in the comparison between SA in solution and solid states (corresponding to the HP-v and HP-s treatments in this study). Based on the above results of viscosity and color, the structural changes of SA treated at 120 °C were selected for a more detailed investigation, aiming to assess the degradation effects and potential structural impacts of autoclaving as a pretreatment method.

#### 2.2.1. Molecular Weight (Mw) of LMWSA Obtained by High-Pressure Treatments

**Figure 4:**
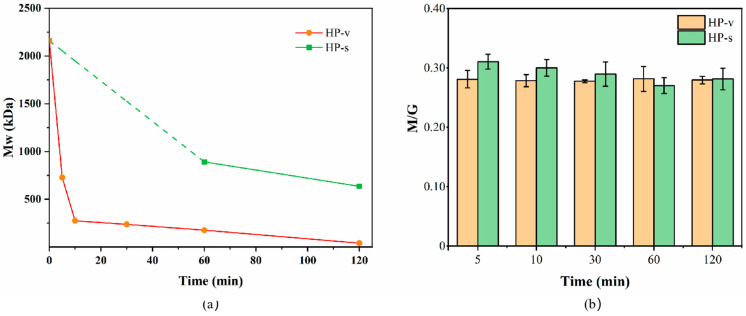
Characteristics of LMWSA obtained by high-pressure treatments: (**a**) Mw distribution (the molecular weight of sodium alginate in HP-s treatment at 120 °C for 5 min, 10 min and 30 min exceeded the range of G4000PWXL analysis column; (**b**) M/G ratio.

The biological activity of SA is contingent upon several essential structural properties, including the type and bonding mode of monosaccharide, the content of active groups, and the Mw of the polysaccharide [5]. Meanwhile, the extent of polysaccharide degradation is typically indicated by alterations in its Mw [34]. As shown in Figure 4a, the variation in the Mw of SA exhibited a trend similar to the decrease in viscosity (see Figure 1). Compared to the original SA, which had an Mw of approximately 2160 kDa, the Mw of LWMSA decreased rapidly with short-term HP-v treatment, dropping to 728.45 kDa, 274.78 kDa, and 40.92 kDa after autoclaving for 5 min, 10 min, and 120 min, respectively. The tendency of Mw changes in the HP-s treatment was similar, exhibiting a higher degradation rate at the initial stage. And the Mw of LMWSA decreased to 635.77 kDa after 120 min of treatment. These changes in Mw further confirmed the degradation effect of SA under high-pressure treatments. Notably, the degradation efficiency of HP-v treatment was higher, with the degradation effect of the HP-v treatment for 10 min being even better than that of the HP-s treatment for 120 min.

Among other physical treatment methods, Watthanaphanit & Saito [26] observed the depolymerization of SA via plasma treatment (SPP) in solution. Following 60 min of SPP treatment, the Mw of SA with concentrations of 0.2%, 0.5% and 0.9% (*w*/*v*) decreased significantly, from 1250 kDa to 130 kDa, 440 kDa and 140 kDa, respectively, corresponding to the 89.6%, 64.8% and 88.8% decreases in the Mw. Burana-osot et al. [35] performed a TiO_2_-catalyzed photochemical reaction to depolymerize SA. The Mw of SA decreased after 3 h of photolysis and more slowly decreased after 6 h of photolysis, which reduced to 108 kDa and 70 kDa, respectively, representing decreases of 45.5% and 64.6% compared to the original polysaccharide. In contrast, the HP-v treatment in this study showed a faster and more efficient degradation effect, with a decrease of 66.3% in the Mw processing at 120 °C for 5 min. However, the Mw of the obtained LMWSA product was still considerably high, necessitating further enzymolysis to prepare AOS with accurate structure and superior biological activity.

#### 2.2.2. M/G Ratio of LMWSA Obtained by High-Pressure Treatments

The properties of SA are strongly influenced by the contents of G and M [6], and the degradation methods may directly lead to alterations in the M/G ratio of SA. For example, under hydrothermal conditions at high temperatures (180–220 °C), M gave higher yields than G at low reaction times, but its yield fell below than that of G with increasing reaction times [24]. Irradiation also affected the degradation products of SA. The M/G ratio of SA diminished with the increasing γ-ray irradiation dose, which reduced noticeably from 1.88 to 0.79 at a dose of 200 kGy [23]. In this study, the change in the M/G ratio of SA at 120 °C under high-pressure treatments is shown in Figure 4b. Compared to the initial SA (0.2737), the M/G ratio of LMWSA did not change significantly with increasing treatment time, remaining around 0.27. This suggested that glycosidic linkages broke under autoclaving processing without a specific preference for G-G, G-M or M-M bonds of SA.

When preparing AOS by enzymolysis, the M/G ratio of the products varies depending on the type of alginate lyase and its specificity for substrate cleavage sites [12]. In this study, both HP-v and HP-s treatments of SA had no effect on its inherent M/G ratio. As a pretreatment for enzymolysis, the monosaccharide composition of the substrate was unchanged by autoclaving treatments, thus not influencing the degradation pattern of the substrate by alginate lyase.

#### 2.2.3. FT-IR Spectra of LMWSA Obtained by High-Pressure Treatments

**Figure 5:**
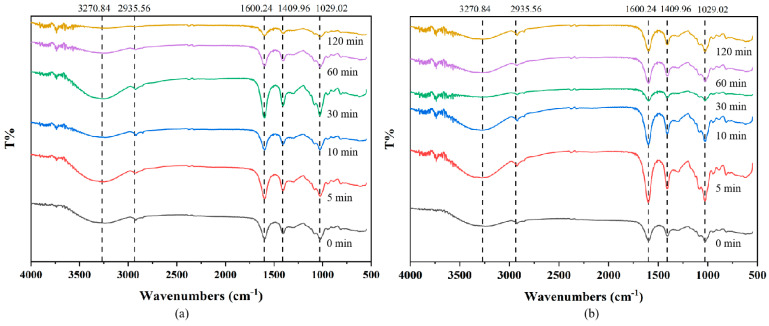
FT-IR spectra of LMWSA obtained by high-pressure treatments at 120 °C: (**a**) HP-v treatment; (**b**) HP-s treatment.

The results of FT-IR spectra of SA in both HP-v and HP-s treatments are shown in Figure 5. Peaks of SA at 3270.84 cm^−1^, 2935.56 cm^−1^ and 1600.24 cm^−1^ were attributed to O-H stretching vibrations, C-H stretching vibrations, and carboxylate O-CO- asymmetric stretching vibrations, respectively. Another two absorption peaks that correspond to C-OH deformation vibration at 1409.96 cm^−1^ and C-O-C tensile vibration at 1029.02 cm^−1^ were observed [36,37]. Peaks at 1600 cm^−1^ for SA were taken as the reference peaks due to the fact that carboxyl would not change after degradation [28]. The scission of glycosidic bonds led to the formation of hydroxyl group, which was manifested as an increase in the ratio of hydroxyl group peak to the reference peak with the simultaneous decreasing of the peak ratio of C-O-C group to the reference, which might be closely related to the degradation of SA.

However, no new band and other measurable changes were observed in the FT-IR spectra of LMWSA after either two media of high-pressure treatments, even at the treatment time of 120 min when the color of LMWSA solution had distinctly turned brown. This indicated that high-pressure treatments did not significantly change the inherent structure of SA, making it a viable option for enzymatic pretreatment.

### 2.3. Analysis of Products After Enzymolysis Procedure

#### 2.3.1. ESI-MS of AOS Products After Enzymolysis Procedure

In order to assess the impact of high-pressure pretreatment on subsequent enzymolysis, LMWSA samples treated for 5 min and 120 min at 120 °C under HP-v and HP-s conditions were subjected to hydrolysis by alginate lyase. The influence of the enzymolysis products from pretreated samples was compared with those from untreated initial SA to validate the potential of autoclaving-assisted enzymolysis.

**Figure 6:**
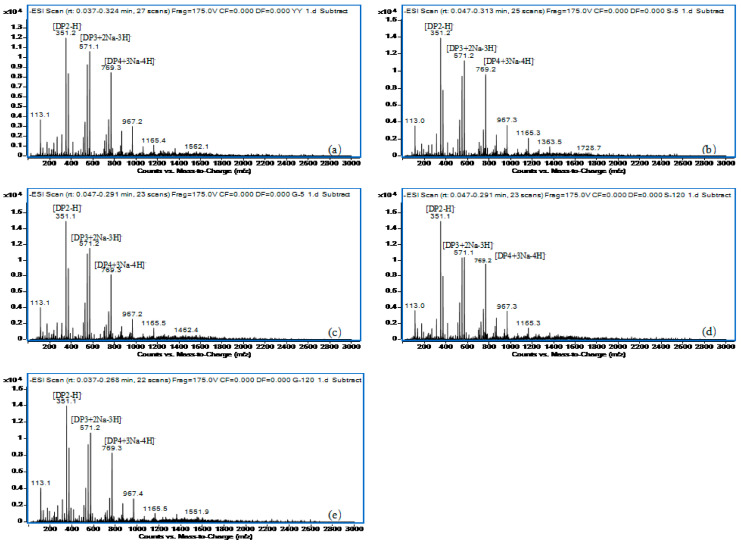
ESI-MS of AOS products after enzymolysis procedure: (**a**) initial SA; (**b**) pretreated SA in HP-v treatment at 120 °C for 5 min; (**c**) pretreated SA in HP-s treatment at 120 °C for 5 min; (**d**) pretreated SA in HP-v treatment at 120 °C for 120 min; (**e**) pretreated SA in HP-s treatment at 120 °C for 120 min.

As shown in Figure 6, the negative-ion mode mass spectra exhibited signals corresponding to the primary deprotonated oligomers [M − H]^−^ (where M represents the MW of the main solute), along with a series of negative ions containing Na, including [M + Na − 2H]^−^, [M + 2Na − 3H]^−^, [M + 4Na − 5H]^−^, etc. [38]. Figure 6a presents the ESI-MS data for the AOS products obtained directly from the enzymolysis of initial SA, while Figure 6b–e show the ESI-MS spectra for AOS samples following pretreatments. The most abundant ion fragment peak at 351 *m*/*z*, [DP2-H]^−^ corresponded to unsaturated disaccharides (C_12_H_15_O_12_). And the ions peaks at 571 and 769 *m*/*z* represented [DP3 + 2Na − 3H]^−^ and [DP4 + 3Na − 4H]^−^, respectively (DP refers to the degree of oligomer polymerization) [39]. The fragment peak at 113 *m*/*z* was presumed to result from the removal of a carboxyl group following the formation of a double bond in the sugar ring of a monosaccharide, i.e., [M1-COOH + H − H]^−^, with M1-158 corresponding to C_5_H_5_O_3_.

The abundance and composition of ion peaks across the aforementioned treatments were highly similar, indicating that the oligosaccharides formed had polymerization degrees ranging from 2 to 6, with disaccharides constituting the highest proportion, followed by trisaccharides and tetrasaccharides, consistent with the information obtained from Yang’s study [40]. Different alginate lyases exhibit distinct specificity and preferences for the bond cleavage process, resulting in AOS products with specific chemical compositions and structures [7]. In this study, the structural information of oligosaccharides derived from the same alginate lyase remained consistent, regardless of whether it undergoes specific high-pressure pretreatments. Furthermore, the final composition of the enzymolysis product was not affected by enzymolysis following high-pressure pretreatments, offering the potential to produce AOS with a definite structure by integrating enzymolysis with autoclaving as a pretreatment.

#### 2.3.2. Mw Development of AOS Products Treated by HP-v Pretreatment During Enzymolysis Procedure

The viscosity results indicated that the degradation effect of high-pressure treatments was superior to that of the AP-a treatment, fulfilling the requirements for pretreatment. The results from FT-IR, ESI-MS, and the M/G ratio of treated SA all confirmed the reliability of high-pressure treatment as a method for enzymolysis. Compared with the HP-v treatment, the HP-s treatment, which was conducted in an aqueous solution medium, was still limited by the large viscosity of SA, less dissolution and low material/liquid ratio during dissolving. In contrast, the HP-v treatment, a direct high-pressure treatment of SA powder, offered more advantages such as greater treatment capacity, higher efficiency, easier storage, and compliance with actual production requirements. As described in Section 2.1, HP-v treatment at 120 °C for 5 min achieved a viscosity reduction of approximately 94% and improved the fluidity of the solution, making it a suitable pretreatment condition for enzymolysis under high substrate concentration. Therefore, this treatment condition was selected to further explore the promotion effect on enzymolysis.

In this study, the concentration of pretreated SA substrate could be elevated to 6% following complete dissolution, with the criterion being the absence of agglomeration and precipitation in the solution system after 24 h of stirring, whereas untreated SA only achieved 2%. Consequently, enzymolysis was performed on the pretreated SA at substrate concentration levels of 2%, 4%, and 6%, and the untreated SA at 2% as a control. Table 1 shows the Mw distribution and proportion of the enzymolysis products. After 60 min of enzymolysis, the products from both initial and pretreated SA with different concentrations exhibited an Mw of approximately 4.0 kDa and 1.4 kDa, in a ratio of about 9:1. This showed that the Mw and its distribution of the products remained consistent upon complete enzymolysis of the SA substrate, further corroborating the findings from the ESI-MS analysis.

The Mw distribution of initial and pretreated SA at a concentration level of 2% after enzymolysis for the same duration reveals that, after 5 min of enzymolysis, the pretreated SA exhibited a higher degree of degradation, and the product composition was characterized by three distinct Mw fractions, namely, 1.42 kDa, 4.20 kDa, and 509 kDa, with respective proportions of 9.95%, 12.79%, and 77.26%, respectively. In contrast, the Mw of unpretreated hydrolysate only concentrated on the component of 1750 kDa, with a low degree of degradation. After 30 min of enzymolysis, 74.33% of the hydrolysate from initial SA had an Mw of approximately 400 kDa, whereas 74.18% of the pretreated SA had an Mw of approximately 117 kDa, with the Mw and distribution of the remaining two fractions being consistent. Consequently, when the proportions of the components were comparable, the Mw of the high molecular weight fraction of the pretreated SA’s degradation products was lower, indicating a higher degree of degradation. After enzymolysis for 60 min, the initial and pretreated SA had undergone full enzymolysis, and the Mw distribution and proportion were similar. HP-v pretreatment could effectively break the main chain of SA and reduce its molecular weight, making it easier for alginate lyase to act on the enzymolysis site.

Comparing the Mw of pretreated SA after enzymolysis at substrate concentrations of 2%, 4%, and 6% for the same duration, the results indicated that after 5 min of enzymolysis, 9.95% of the products at 2% substrate concentration had an Mw of approximately 1.4 kDa, while the percentages at 4% and 6% substrate concentrations were 3.71% and 5.01%, respectively, aligning with the expected trend of enzymatic action. Interestingly, the Mw of the predominant component at 2% substrate concentration was approximately 509 kDa, with 235 kDa at 4% and 88 kDa at 6%, indicating a more effective enzymolysis at higher substrate concentrations. This phenomenon could be attributed to the fact that the high substrate concentrations provide more substrate molecules simultaneous interaction with the alginate lyase, leading to more comprehensive interactions and allowing the enzyme’s potential to be fully realized. It was consistent with the classical kinetic theory of enzyme-catalyzed reactions, suggesting that, within a certain range, an increase in substrate concentration would enhance reaction rates [41]. In addition, the Mw changes observed throughout the enzymolysis process suggested that the pretreated SA at a 6% substrate concentration not only accelerated the enzymolysis process but also achieved complete enzymolysis sooner. It reflected indirectly that enzyme function was constrained by substrate concentration, and its full potential was not easily realized at low concentrations. The advantage of HP-v pretreatment in promoting enzymolysis also reflected the better utilization of alginate lyase activity.

## 3. Materials and Methods

### 3.1. Materials

SA used in the present experiment, with an M/G ratio of 0.2737 and a Mw of 2160 kDa, was purchased from Sinopharm Chemical Reagent Co., Ltd. (Shanghai, China). Alginate lyase was provided by the Laboratory of Marine Microbiological Engineering Laboratory at the Ocean University of China [42]. Ultra-pure water from the Aquarius water distillation apparatus, RFD250NB, Advantec (Tokyo, Japan), was used for the preparation of all alginate aqueous solutions. And all other commercial reagents were of analytical grade.

### 3.2. Preparation of LMWSA

SA powder (8 g) was suspended in 400 mL of distilled water (pH 7.0) on a magnetic stirrer at 25 °C to prepare an SA aqueous solution with a concentration of 0.02 g/mL. The prepared SA aqueous solution was subjected to high-pressure heating in an autoclave (YXQ-LS-50S11, Shanghai Boxun Industrial and Commercial Co., Ltd., Shanghai, China), designated as the “HP-s” treatment group.

An equivalent amount of SA powder, contained within 100-mesh vent bags to ensure uniform depolymerization, was heated in water vapor under high pressure in the autoclave, and this treatment was designated as the “HP-v” treatment group.

The SA powder, prepared using the same method as the HP-v treatment, was heated in a laboratory oven (LDO-101-2, Shanghai Yue Long Instrument Co., Ltd., Shanghai, China) under atmospheric pressure and designated as the “AP-a” treatment group.

SA treated in the aforementioned three specific media was heated at 110 °C, 115 °C, 120 °C and 125 °C for 2 h, with samples of LMWSA collected at 5 min, 10 min, 30 min, 60 min and 120 min. The corresponding steam pressures of the HP-s and HP-v treatment groups at different temperatures were 0.042 MPa, 0.07 MPa, 0.1 MPa, and 0.135 MPa, respectively. Finally, all samples were lyophilized at −80 °C and 5 Pa for further experiments using a freeze dryer (FD-1A, BIOCOOL, Beijing, China).

### 3.3. Enzymolysis Procedure of SA and LMWSA

Next, 100 mL of SA and LMWSA solution with concentrations of 2%, 4% and 6% were prepared, and alginate lyase with substrate content of 50% was added for water bath enzymolysis at 55 °C, pH 7.0. The reaction was terminated by heating at 80 °C for 10 min after enzymolysis for 5 min, 10 min, 15 min, 20 min, 30 min and 60 min. The hydrolysate was centrifuged at 4000 rpm for 10 min, and the supernatant was reserved for analysis.

### 3.4. Characterization

#### 3.4.1. Viscosity of SA and LMWSA

Viscosity measurements were conducted at 20 °C with 1% concentration sample solution (*w*/*v*) using a Digital Viscometer (NDJ-5S, Shanghai Yueping Instrument Co., Ltd., Shanghai, China).

#### 3.4.2. Color of SA and LMWSA

Color changes of the samples at the same concentration were determined by a colorimeter (LS173B, Shenzhen Linshang Technology Co., Ltd., Shenzhen, China). ∆L was calculated as the difference of L values between the initial and processed SA.

#### 3.4.3. UV-Vis Spectra Analysis

The UV-vis spectra of SA solutions were recorded at 25 °C using an UVe3600 spectrophotometer (Shimadzu, Kyoto, Japan) over the wavelength range of 250–500 nm. The concentration of SA solution used for the detection was 0.5% *w*/*v*.

#### 3.4.4. FT-IR Spectra Analysis

FT-IR spectra were collected using a Nicolet iS10 FT-IR spectrometer (Thermo Fisher Scientific, Waltham, MA, USA) at a wavelength range from 4000 to 400 cm^−1^. Each 2–3 mg SA sample was prepared for measurement by grinding with 200–300 mg spectrum-pure anhydrous KBr, followed by compression into discs. The spectral resolution was 4 cm^−1^ with a repetition of 64 scans.

#### 3.4.5. M/G Ratio of SA and LMWSA

The content of M and G was determined by PMP-HPLC with Pre-column Derivatization using mannuronate monosaccharide and guluronate monosaccharide as standards. The M/G ratio was then calculated according to Dai et al. [43].

#### 3.4.6. Molecular Weight of SA and LMWSA

Molecular weight (Mw) of the degradation products was determined by HPLC (Agilent 1260 Infinity HPLC system, Santa Clara, CA, USA) equipped with a refractive index detector (RID) using a TSK gel G4000PWXL column. The mobile phase was NaNO_3_ (200 mM) with 10 mM NaH_2_PO_4_ maintained at 0.5 mL/min, and the concentration of precipitates was adjusted to 0.1% (*w*/*v*) [28]. A series of dextran standards (1500 Da, 3650 Da, 5000 Da, 12,000 Da, 21,000 Da, 80,000 Da and 150,000 Da) were used for the calibration of retention time and calculation.

#### 3.4.7. ESI-MS Analysis

The degradation products were analyzed by negative ion ESI-MS (Agilent 1290 Infinity II-6460, Frag = 175.0 V, *m*/*z* 100–2000 amu, Santa Clara, CA, USA). Each sample was dissolved in water and diluted in 50% aqueous methanol [40].

### 3.5. Statistical Analysis

All analyses were performed on three separate preparations, and the data were presented as the mean ± standard deviation for each sample point. The analysis was performed using least significant difference (LSD) with SPSS software (v.22, IBM SPSS Statistics, Chicago, IL, USA) at a significance level of *p* < 0.05.

## 4. Conclusions

At present, the enzymatic preparation of AOS faces issues including high viscosity and low concentration of substrate, limiting the efficiency of enzymolysis. This study proposed a process design for viscosity reduction pretreatment, screened the pretreatment medium, optimized the conditions, and investigated the structural changes in SA during pretreatment as well as the feasibility of its assistant enzymolysis. The results indicated that HP-v treatment carried out by autoclaving was more effective in reducing viscosity than HP-s and AP-a treatments, achieving a 94% reduction in SA viscosity at 120 °C for 5 min. Subsequently, the potential structural and depolymerization effects on SA under high-pressure treatments were elucidated. ESI-MS analysis confirmed that the structure of AOS derived from enzymolysis was unaffected by high-pressure treatments. However, the underlying mechanisms governing high pressure-induced color changes and their implications for product activity remain to be fully elucidated. To summarize, the brief HP-v treatment could facilitate the preparation of AOS with specific molecular weights, which was achieved by increasing substrate concentration and maximizing the activity of alginate lyase, offering a novel approach and direction for AOS preparation and enhancing the enzymolysis efficiency of alginate lyase.

## Figures and Tables

**Table 1:** Mw composition and distribution of enzymolysis products at different times.

Substrate	Concentration (%)	Enzymolysis Time (min)	Composition and Distribution of Mw					
			Mw_1_ (kDa)	Proportion (%)	Mw_2_ (kDa)	Proportion (%)	Mw_3_ (kDa)	Proportion (%)
Initial SA	2	5	1750.35	100.00	——		——	
		10	1677.65	70.49	4.13	14.78	1.41	14.73
		15	1183.18	71.58	4.02	14.55	1.40	13.87
		20	539.45	75.76	4.08	14.23	1.41	10.01
		30	400.50	74.33	3.98	17.71	1.40	7.96
		60	——		4.02	91.59	1.40	8.41
Pretreated SA	2	5	508.74	77.26	4.20	12.79	1.41	9.95
		10	297.18	75.28	4.12	14.07	1.40	10.65
		15	156.17	73.89	4.12	16.27	1.40	9.84
		20	130.63	79.10	4.08	13.11	1.39	7.79
		30	117.23	74.18	4.05	17.90	1.40	7.92
		60	——		3.99	91.84	1.40	8.16
	4	5	235.26	90.64	4.32	5.66	1.42	3.71
		10	132.12	87.79	4.24	7.36	1.41	4.86
		15	76.97	79.74	4.16	14.10	1.41	6.17
		20	44.67	77.39	4.08	17.76	1.40	4.86
		30	17.14	52.15	4.04	40.04	1.41	7.81
		60	——		4.01	91.89	1.40	8.81
	6	5	88.24	87.33	4.60	7.66	1.41	5.01
		10	58.51	78.66	4.32	15.26	1.42	6.08
		15	40.85	73.53	4.07	19.82	1.41	6.65
		20	——		4.02	90.57	1.42	9.43
		30	——		4.02	91.95	1.41	8.05
		60	——		3.99	90.26	1.41	9.74

## Data Availability

Data will be made available on request.
